# UCP2 modulates single-channel properties of a MCU-dependent Ca^2+^ inward current in mitochondria

**DOI:** 10.1007/s00424-015-1727-z

**Published:** 2015-08-16

**Authors:** Alexander I. Bondarenko, Warisara Parichatikanond, Corina T. Madreiter, Rene Rost, Markus Waldeck-Weiermair, Roland Malli, Wolfgang F. Graier

**Affiliations:** Institute of Molecular Biology and Biochemistry, Center of Molecular Medicine, Medical University of Graz, Harrachgasse 21/III, 8010 Graz, Austria

**Keywords:** Mitochondrial Ca^2+^ channels, Mitochondrial Ca^2+^ currents, Mitochondrial Ca^2+^ uniporter, Mitoplast, Uncoupling protein 2, Patch clamp

## Abstract

The mitochondrial Ca^2+^ uniporter is a highly Ca^2+^-selective protein complex that consists of the pore-forming mitochondrial Ca^2+^ uniporter protein (MCU), the scaffolding essential MCU regulator (EMRE), and mitochondrial calcium uptake 1 and 2 (MICU1/2), which negatively regulate mitochondrial Ca^2+^ uptake. We have previously reported that uncoupling proteins 2 and 3 (UCP2/3) are also engaged in the activity of mitochondrial Ca^2+^ uptake under certain conditions, while the mechanism by which UCP2/3 facilitates mitochondrial Ca^2+^ uniport remains elusive. This work was designed to investigate the impact of UCP2 on the three distinct mitochondrial Ca^2+^ currents found in mitoplasts isolated from HeLa cells, the intermediate- (*i-*), burst- (*b-*) and extra-large (*xl-*) mitochondrial/mitoplast Ca^2+^ currents (MCC). Using the patch clamp technique on mitoplasts from cells with reduced MCU and EMRE unveiled a very high affinity of MCU for *xl-*MCC that succeeds that for *i-*MCC, indicating the coexistence of at least two MCU/EMRE-dependent Ca^2+^ currents. The manipulation of the expression level of UCP2 by either siRNA-mediated knockdown or overexpression changed exclusively the open probability (NPo) of *xl-*MCC by approx. 38 % decrease or nearly a 3-fold increase, respectively. These findings confirm a regulatory role of UCP2 in mitochondrial Ca^2+^ uptake and identify UCP2 as a selective modulator of just one distinct MCU/EMRE-dependent mitochondrial Ca^2+^ inward current.

## Introduction

After decades of research, great progress in the molecular identification of the proteins responsible for mitochondrial Ca^2+^ uptake has been achieved recently. Notably, following the landmark discovery of mitochondrial calcium uptake 1 (MICU1) by the group of Vamsi K. Mootha in the year 2010 [27], further components of the protein complex that achieves mitochondrial Ca^2+^ sequestration have been identified: the mitochondrial Ca^2+^ uniporter (MCU) [1, 10], MICU1, MICU2, and MICU3 [28], and the essential MCU regulator (EMRE) [32]. In our previous work, a functional importance of UCP2/3 for mitochondrial Ca^2+^ uptake upon intracellular Ca^2+^ release but not entering Ca^2+^ was described [36, 38, 40]. As in some subsequent reports these findings could not be supported [19] or the effect of UCP2/3 was claimed to be due to alternative function [8], the exact contribution of UCP2/3 to mitochondrial Ca^2+^ is still unclear [26]. Notably, our data on the role of UCP2/3 in mitochondrial Ca^2+^ uptake indicate a regulatory function of UCP2/3 on MCU-dependent mitochondrial Ca^2+^ uniporter under certain conditions while a direct involvement of UCP2/3 as part of the actual Ca^2+^ pore appears unlikely [15, 35].

Besides the great progress in the identification of proteins and currents that are responsible for mitochondrial Ca^2+^ uptake, the understanding of the molecular regulation of mitochondrial Ca^2+^ uptake has been also significantly grown. Most importantly, MICU1 and MICU2 that together set that Ca^2+^ sensitivity of MCU [7, 20] have been found to (cooperative) negatively regulate mitochondrial Ca^2+^ uptake [22, 25, 28]. Under basal cytosolic/intermembrane Ca^2+^ levels, MICU1 assembles in hexamers [41] and together with MICU2 prevents Ca^2+^ flux via the MCU/EMRE complex [20, 22]. Upon elevation of cytosolic/intermembrane Ca^2+^, Ca^2+^ binds to the two EF hands of MICU1 resulting in disassembly of the MICU1 multi/hexamers [41, 42] and, thus, the release of MCU/EMRE from the MICU1/MICU2 inhibition. Besides the control by MICU1/MICU2, MCU-dependent mitochondrial Ca^2+^ is further controlled on the transcriptional level of MCU expression [14, 33], the proximity to the endoplasmic reticulum [29, 43] and by CaMKII [12]. Other mechanisms of regulation of MCU/EMRE-established mitochondrial Ca^2+^ uptake as utmost important phenomenon controlling mitochondrial activity, malfunction, and ultimately cell death await further investigations [5].

After decades of intense investigations, mitochondrial Ca^2+^ uptake can now be associated with certain proteins that form a protein complex that establishes the well-known mitochondrial Ca^2+^ uptake phenomenon [9, 21, 42]. Aside the discovery of proteins that establish mitochondrial Ca^2+^ uptake, heart-specific ryanodine receptor-dependent Ca^2+^ currents [2, 16, 30, 31], the Leucine zipper/EF hand containing transmembrane protein 1 (Letm1)-dependent Ca^2+^ fluxes [18] and several putative Ca^2+^ uniporter Ca^2+^ currents [3, 17, 23] have been electrophysiologically described in mitoplasts (for review, see [30, 34]). Some of these channels could be ascribed to MCU [4, 6] though a final judgment which of the described mitoplast Ca^2+^ currents represents the actual mitochondrial Ca^2+^ uptake in intact cells cannot be given so far.

In view of the yet unresolved contribution of UCP2/3 to mitochondrial Ca^2+^ uptake, this work was designed to explore the contribution of UCP2 to mitochondrial Ca^2+^ currents. Therefore, the impact of a diminution as well as overexpression of UCP2 on the occurrence and biophysical characteristics of mitochondrial/mitoplast Ca^2+^ currents was evaluated using HeLa cells that have been found to exhibit UCP2-modulated mitochondrial Ca^2+^ uptake [36] and three distinct mitoplast Ca^2+^ inward currents [3, 4, 17].

## Methods

### Cell culture and isolation of mitochondria

All cells were grown on DMEM containing 10 % FCS, 50 U/ml penicillin, and 50 μg/ml streptomycin. Mitochondria were freshly isolated as previously described [3, 4]. Mitochondria were prepared from HeLa cells by differential centrifugation. Cells were trypsinized, harvested, and washed with PBS. The cell pellet was suspended in a 200 mM sucrose buffer containing 10 mM Tris-MOPS, 1 mM EGTA, and protease inhibitor (1:50, P8340 Sigma, Vienna, Austria) (pH adjusted to 7.4 with TRIS) and homogenized with a glass–Teflon potter (40–50 strokes). Nuclear remnants and cell debris were centrifuged down at 900*g* for 10 min. The supernatant was centrifuged at 3000*g* for 20 min. The mitochondrial pellet was washed and centrifuged down at 7000*g* for 15 min. All fractions were kept on ice until further utilization.

### Design and production of stably MCU knockdown HeLa cells and their corresponding control cells

HeLa MCU KD and HeLa control cells have been produced upon request and supplied by TeBu-bio^®^ (tebu-bio SAS, Le Perray-en-Yvelines Cedex, France) and previously described [4]. HeLa cells with stable MCU knockdown and the respective scrambled control cells were produced by applying the SilenciX^®^ technology (Tebu-bio, www.tebu-bio.com, Le Perray-en-Yvelines, France) using the following 5′–3′shRNA sequence against MCU: GGTGCAATTTATCTTTATA. Using quantitative real-time PCR, the efficiency of stably MCU knockdown was 73.4 ± 1.0 % in this particular cell type.

### Specific siRNAs

For silencing hMCU or hEMRE, we used siRNAs from Microsynth (Balgach, Switzerland) with following sequences: (sense strands, 5′–3′): hMCU-si1 (GCCAGAGACAGACAAUACU), hMCU-si2 (GGAAAGGGAGCUUAUUGAA); hEMRE-si (GAACUUUGCUGCUCUACUU).

### Quantitative real-time PCR

We used the PEQLAB total RNA isolation kit (PEQLAB Biotechnologie GmBH, Erlangen, Germany) for total RNA isolation. RNA samples (1 μg each) were reverse-transcribed with the cDNA synthesis kit (Applied Biosystems, USA). Efficiency of knockdown was assessed with a LightCycler 480 (Roche Diagnostics, Vienna, Austria). As housekeeping gene human, GAPDH (no. QT01192646, QuantiTect^®^ Primer Assay, Qiagen, Hilden, Germany) was used. Target genes were amplified using the GoTaq^®^ qPCR Master Mix (Promega) and specific real-time primer pairs (Invitrogen): hMCU forward 5′-TTCCTGGCAGAATTTGGGAG-3′, hMCU reverse 5′-AGAGATAGGCTTGAGTGTGAAC-3′; hEMRE forward 5′-TCGCTGGCTAGTATTGGCAC-3′, hEMRE reverse 5′-GGAGAAGGCCGAAGGACATT-3′. Relative expression of the hEMRE and hMCU were normalized to GAPDH expression and analyzed by the REST software (Qiagen, Hilden, Germany).

### Knockdown efficiencies for MCU, EMRE, and UCP2

Further transient transfection of stably MCU knockdown cells with a previously approved siRNA against MCU [11] yielded an overall 80.7 ± 0.7 % (*n* = 3) reduction. Efficiency of siRNA against EMRE in the stably MCU knockdown cells was evaluated with quantitative RT-PCR and revealed a reduction by 46.6 ± 3.3 % (*n* = 3) of this particular protein. Diminution in UCP2 gene expression in HeLa cells using the respective and previously approved siRNA [40] was confirmed by quantitative real-time PCR to be depleted by 84.4 ± 1.1 % (*n* = 3) of the level detected in control cells.

### Preparation of mitoplasts

Isolation and preparation of mitoplasts (mitochondria devoid of outer membrane) from HeLa cells was performed as recently described [3]. Briefly, mitoplast formation was achieved by incubation of isolated mitochondria in hypotonic solution (5 mM HEPES, 5 mM sucrose, 1 mM EGTA, pH adjusted to 7.4 with KOH) for 8 min. Then, hypertonic solution (750 mM KCl, 80 mM HEPES, 1 mM EGTA, pH adjusted to 7.4 with KOH) was added to restore isotonicity.

### Mitoplast patch clamp recordings

Single-channel measurements were performed in the mitoplast-attached configuration as previously described [3, 4, 17]. In brief, patch pipettes were pulled from glass capillaries using a Narishige puller (Narishige Co., Ltd., Tokyo, Japan), fire-polished and had a resistance of 8–12 MΩ. Mitoplasts were bathed in the solution containing the following (in mM): 145 KCl, 1 EGTA, HEPES, pH adjusted to 7.2 with KOH. For single-channel recordings, the pipette solution contained 105 mM CaCl_2_ and 10 mM HEPES, 10 μM cyclosporin A (Tocris Bioscience, Bristol, UK) and 10 μM 7-chloro-5-(2-chlorophenyl)-1,5-dihydro-4,1-benzothiazepin-2(3*H*)-one (CGP 37157, Ascent Scientific Ltd., Bristol, UK) to prevent opening of the permeability transition pore (PTP), and the activity of the mitochondrial Na^+^/Ca^2+^ exchanger (NCX_mito_), respectively. pH was adjusted to 7.2 with Ca(OH)_2_. Single-channel currents were recorded at a fixed holding potential indicated in the respective figures. For whole-mitoplast recordings, pipette solution contained the following (in mM): 120 CsMethanesulfonate, 30 CsCl, 1 EGTA, 110 sucrose, 2 gluconic acid, pH by TEAOH to 7.2. For obtaining whole-mitoplast configuration, voltage steps of 300–600 mV and 20–50-ms duration were applied. Voltage ramps of 1-s duration from −160 to +50 mV were delivered every 5 or 10 s from the holding potential 0 mV. Currents were recorded using a patch clamp amplifier (EPC7, List Electronics, Darmstadt, Germany). Data collection was performed using Clampex software of pClamp (V9.0, Molecular Devices, Sunnyvale, CA, USA). Signals obtained were low pass filtered at 1 kHz using an eight-pole Bessel filter (Frequency Devices), and digitized with a sample rate of 10 kHz using a Digidata 1200A A/D converter (Molecular Devices, Sunnyvale, CA, USA). All measurements were performed at room temperature. For recording cationic currents via whole mitoplasts, bath solution contained the following (in mM): 150 TRIS HCl, 1 EGTA, 1 EDTA, 10 HEPES with pH 7.2. For I_Na_ recording, NaCl was substituted for TRIS HCl. Ca^2+^-containing bath solution for I_Ca_ recording contained (in mM): 140 TRIS HCl, 3 CaCl_2_, 10 HEPES, pH 7.2.

### Statistical analysis

The occurrence probability was calculated as a fraction of patches displayed specific channel activity relative to the total number of patches studied in a given experimental day (N^D^). The number of patches studied per day varied from 2 to 12. Mean values of occurrence probability of specific channel activity were derived from respective individual values reflecting respective occurrence in a given experimental day. Single-channel analysis was performed using Clampfit 9.2 (Molecular Devices, Sunnyvale, CA, USA). Data are expressed as mean values with standard error. Statistical comparisons were conducted with a two-tailed unpaired *t* test. Values of *P* < 0.05 (*) were taken as statistically significant. Statistical analysis was performed by GraphPad Software version 5.01 (La Jolla, CA, USA). As an index of steady-state channel activity, we used the product of the number of channels in the patch during recording (N) and the open channel probability (Po). NPo was obtained using a built-in option in Clampfit 9.2 (Molecular Devices) from ≥20 s of recording.

## Results

### UCP2 does not form new Ca^2+^ channels but affects the occurrence of *i*-MCC and *xl-*MCC without affecting that of *b-*MCC

**Fig. 1:**
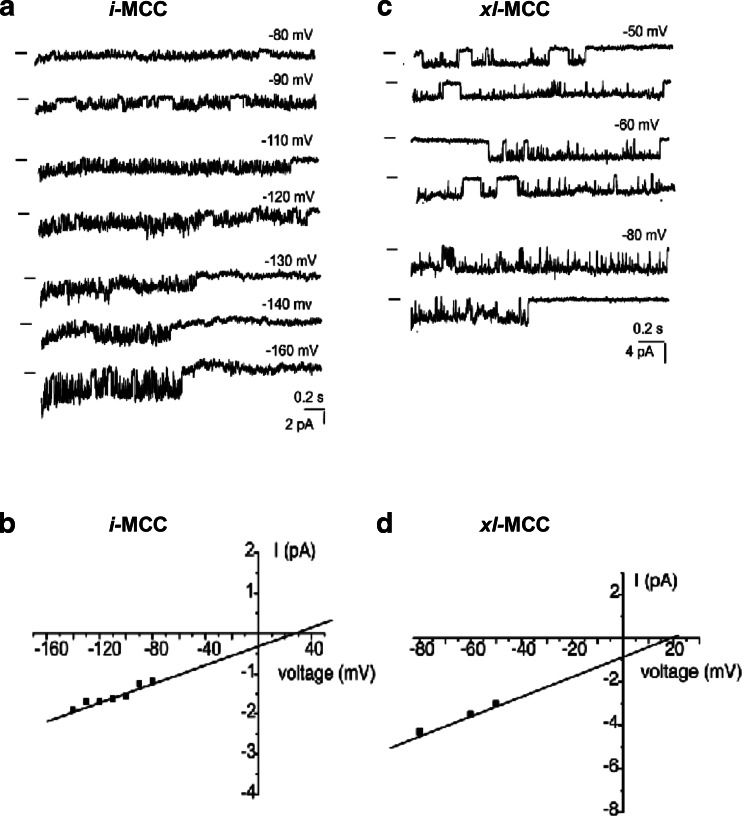
Presentation of *i-*MCC and *xl*-MCC activities in isolated mitoplasts. **a** Representative traces of i-MCC activity at different voltages. Closed states are indicated by *bars*. **b** Corresponding current-voltage relationship of *i*-MCC. **c** Representative traces of *xl*-MCC activity at different voltages. Closed states are indicated by *bars*. **d** Corresponding current-voltage relationship of *xl*-MCC

**Fig. 2:**
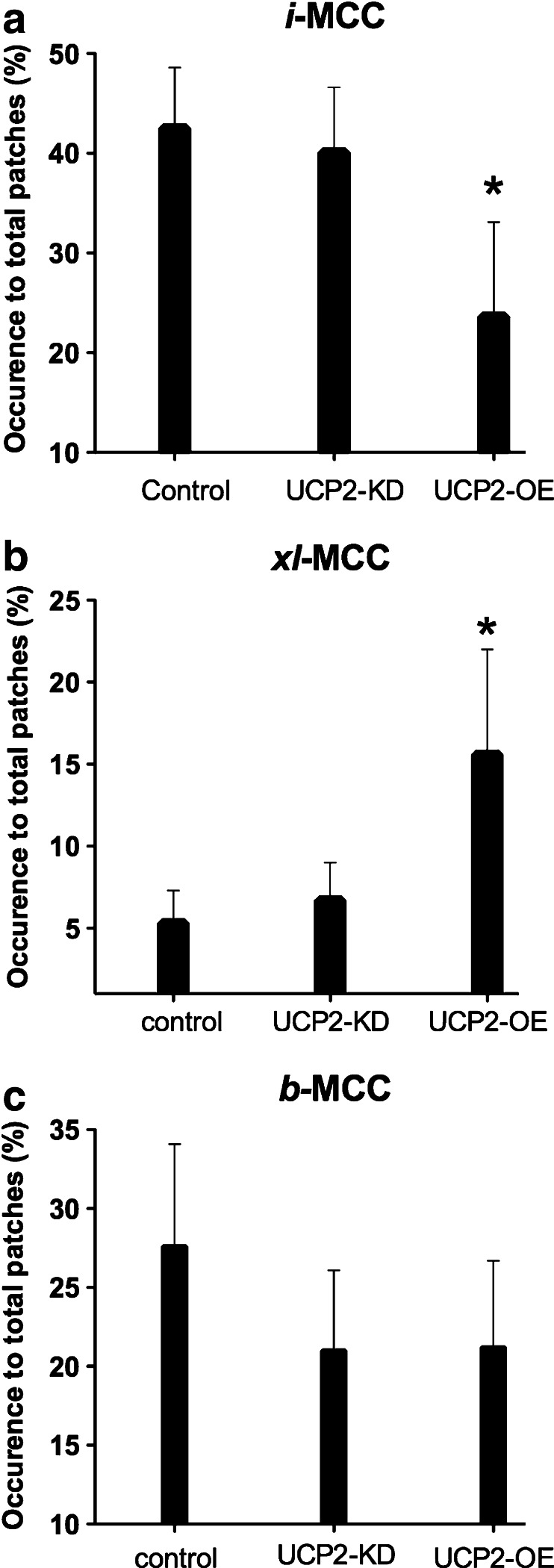
The effect of manipulations in the level of UCP2 expression on occurrence of distinct single channel activities in the inner mitochondrial level of HeLa cells. **a** *Bars* represent the mean level of *i*-MCC occurrence in control, UCP2-KD, and UCP2-overexpressed groups. **b** *Bars* represent the mean level of *xl*-MCC occurrence in control, UCP2-KD, and UCP2-overexpressed groups. **c** *Bars* represent the mean level of *b*-MCC occurrence in control, UCP2-KD, and UCP2-overexpressed groups. *P < 0.05 vs. control

In mitoplasts isolated from control cells, UCP2-overexpressing cells and UCP2-KD cells, the active patch frequency amounted 70.9 % (88 active out of 124 tested patches), 47.5 % (47 active out of 99 tested patches), and 50.4 % (57 active out of 113 tested patches), respectively. Previously, we described single-channel properties of the three different channel populations with distinct unitary conductances *i-*MCC, *b-*MCC, and *xl-*MCC [3]. Representative traces of *i*-MCC and *xl*-MCC at different voltages are shown in Fig. 1. To verify whether or not UCP2 forms new Ca^2+^-permeable channels in the inner mitochondrial membrane, analyses of individual Ca^2+^ conductance in mitoplasts of UCP2-overexpressing cells were compared with those from respective control cells. In both groups, the already described three distinct Ca^2+^ conductances, *i-*MCC, *xl-*MCC, and *b-*MCC [3] were found. UCP2 overexpression did not result in the appearance of a further Ca^2+^ conductance in mitoplasts, thus excluding the possibility of UCP2 as core protein for a Ca^2+^ channel in the inner mitochondrial membrane. Further analyses of the proportion of each individual channel activity was tested by comparing the mean occurrence probability of each individual channel activity for each individual experimental day and calculating statistics out of the individual values from all experimental days (N^D^) [4]. Notably, overexpression of UCP2 reduced the occurrence of *i-*MCC by 44 % from 42.5 ± 6.1 % (54 patches among 124 tested N^D^ = 37) in controls to 23.6 ± 6.3 % (19 patches among 99 tested N^D^ = 17; *P* < 0.05) in mitoplasts isolated from UCP2-overexpressing cells (Fig. 2a). In contrast, the occurrence of *xl-*MCC increased from 5.3 ± 2.9 % in mitoplasts from control cells (9 patches among 124 tested, N^D^ = 37) to 15.6 ± 6.4 % (12 patches out of 99 tested N^D^ = 17; *P* = 0.14) in mitoplasts from UCP2-overexpressing cells (Fig. 2b). Occurrence of *b-*MCC remained unaltered by UCP2 overexpression (Fig. 2c). These data indicate that largely increased amounts of UCP2 shifts the appearance of individual mitoplast Ca^2+^ conductances in favor for *xl-*MCC at the cost of the appearance of *i*-MCC.

We next explored the impact of UCP2 knockdown on the occurrence of *i-*MCC, *b-*MCC, and *xl-*MCC. The *i-*MCC, *xl-*MCC, and *b-*MCC were detected in 40.1 ± 6.5, 6.7 ± 2.3, and 21.0 ± 5.1 % of mitoplast recordings from UCP2-KD cells, respectively (N^D^ = 17) (Fig. 2a–c). Due to the general limitations of statistical analysis due to uneven number of successful patches studied per day which results in a large variation in relation to the actual low number 5–6 % in the occurrence of *xl-*MCC, a statistical evaluation of the knockdown data appear not appropriate. Thus, a clear outcome whether or not downregulation of UCP2 affects the probability of occurrence of *xl-*MCC in terms of total number of patches studied or to a number of active patches cannot be provided yet.

### *xl-*MCC but not *i-*MCC is regulated by UCP2

**Fig. 3:**
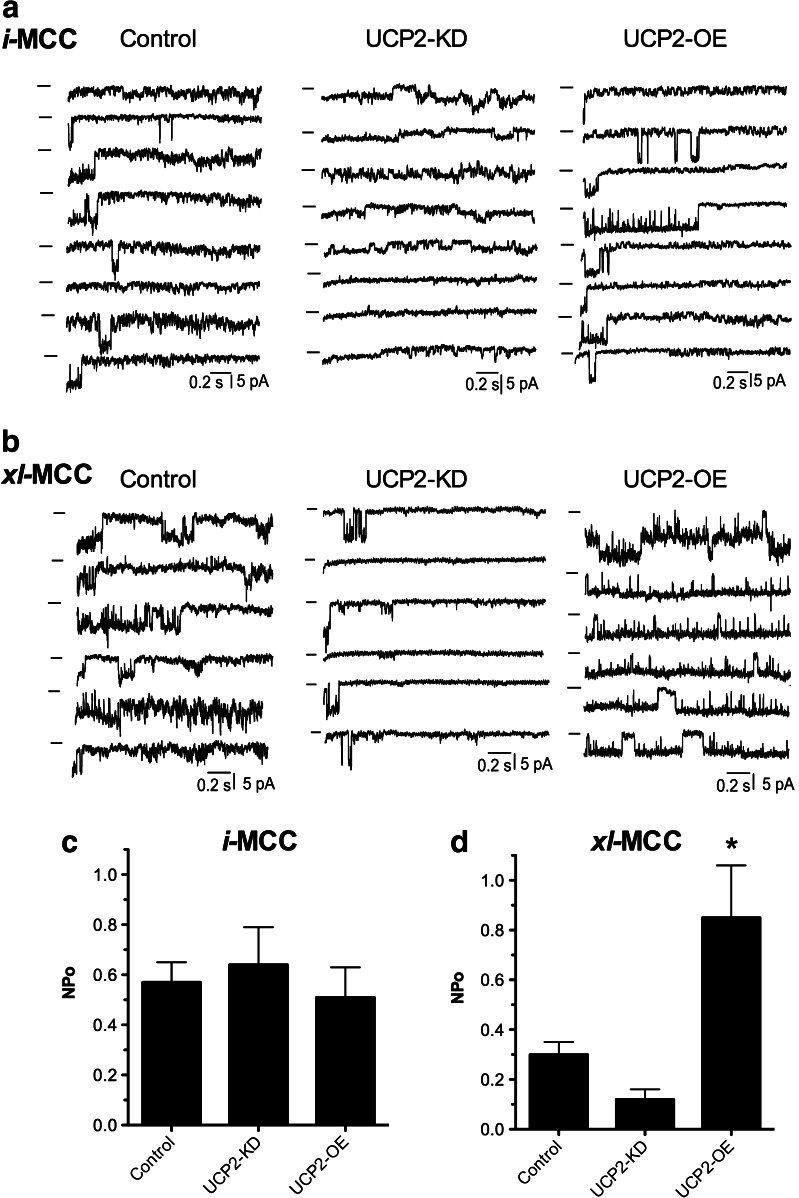
Single-channel recordings showing distinct channel activities in cell lines with variable levels of UCP2 expression. **a** Exemplary traces showing *i*-MCC activities in control (*left*), UCP2-overexpressed (*middle*), and UCP2 knockdown (*right*) groups. In traces from control and UCP2-overexpressed group, the *i*-MCC activity is interrupted by *xl*-MCC activity represented by single-channel opening of higher amplitude. Pipette potential is 100 mV. Channel openings are presented as downward eflections. Closed states are indicated by *bars*. **b** Exemplary traces showing *xl*-MCC activities in control (*left*), UCP2-overexpressed (*middle*), and UCP2 knockdown (*right*) groups. Pipette potential is 100 mV. Channel openings are presented as downward deflections. Closed states are indicated by *bars*. **c** Statistical presentation of mean NPo values of *i*-MCC in control, UCP2-KD, and UCP2-overexpressed group. **d** Statistical presentation of mean NPo values of *xl*-MCC in control, UCP2-KD, and UCP2-overexpressed group. *P < 0.05 vs. control

Next, we analyzed whether the level of UCP2 expression affects the open probability (NPo) of *i-*MCC and *xl-*MCC. The NPo of *i-*MCC was unaffected in both UCP2-KD and UCP2-overexpressing cells (control, 0.62 ± 0.07, *n* = 32; UCP2-KD, 0.62 ± 0.14, *n* = 13; UCP2 overexpression, 0.51 ± 0.14, *n* = 14) (Fig. 3a, c). Additionally, the conductance of *i-*MCC remained unchanged by UCP knockdown (control, 12.9 ± 0. 7, *n* = 13; UCP2-KD, 13.2 ± 0.8 pS, *n* = 14; UCP2 overexpression, 11.6 ± 0.6, *n* = 19).

In mitoplasts isolated from UCP2-overexpressing cells, NPo of *xl-*MCC was largely increased by 293 % from 0.29 ± 0.06 (*n* = 9) in control mitoplasts to 0.85 ± 0.21 (*n* = 7; *P* < 0.05) in mitoplasts isolated from UCP2-overexpressing cells. Moreover, in mitoplasts prepared from UCP2-KD cells, the mean NPo value of *xl-*MCC was reduced by 38 % compared to control to 0.18 ± 0.06 (*n* = 10) (Fig. 3b, d). In contrast to the NPo of the *xl-*MCC, its mean conductance was not significantly different between the three groups (control, 65.8 ± 4.6 pS, *n* = 10; UCP2-KD, 75.2 ± 4.0 pS, *n* = 10; UCP2 overexpression, 51.0 ± 5.5 pS, *n* = 7). These data point to an exclusive regulatory function of UCP2 on *xl-*MCC but not *i-*MCC.

### Combined MCU and EMRE downregulation revealed *xl-*MCC but not *b-*MCC to depend on these both proteins

In our previous work using MCU knockdown cells, only the appearance of *i-*MCC but not that of *xl-*MCC and *b-*MCC was reduced [4]. In fact, while the abundance of *i-*MCC was strongly reduced by moderate MCU knockdown (i.e., by 36 ± 10 and 33 ± 6 % of the respective mRNA and protein, respectively), abundance of *xl*-MCC actually increased by 2.3-fold [4], thus indicating some interrelation between *i-*MCC and *xl-*MCC. To assess the link between *xl-*MCC activity and expression of MCU and EMRE, the two major components of the mitochondrial Ca^2+^ uniporter [9, 21], we analyzed whether downregulation of MCU and EMRE influences the occurrence probability of each individual Ca^2+^ conductance found in mitoplasts. In MCU-KD cells additionally treated with siRNA against MCU and EMRE (MCU-KD/siMCU-siEMRE), the occurrence of active patches with any channel activity decreased by 40 % compared with untreated cells from 71.4 ± 5.7 % (88 patches out of 124 tested, N^D^ = 32) to 42.3 ± 7.5 % (43 patches out of 85 tested, N^D^ = 16; *P* < 0.05).

**Fig. 4:**
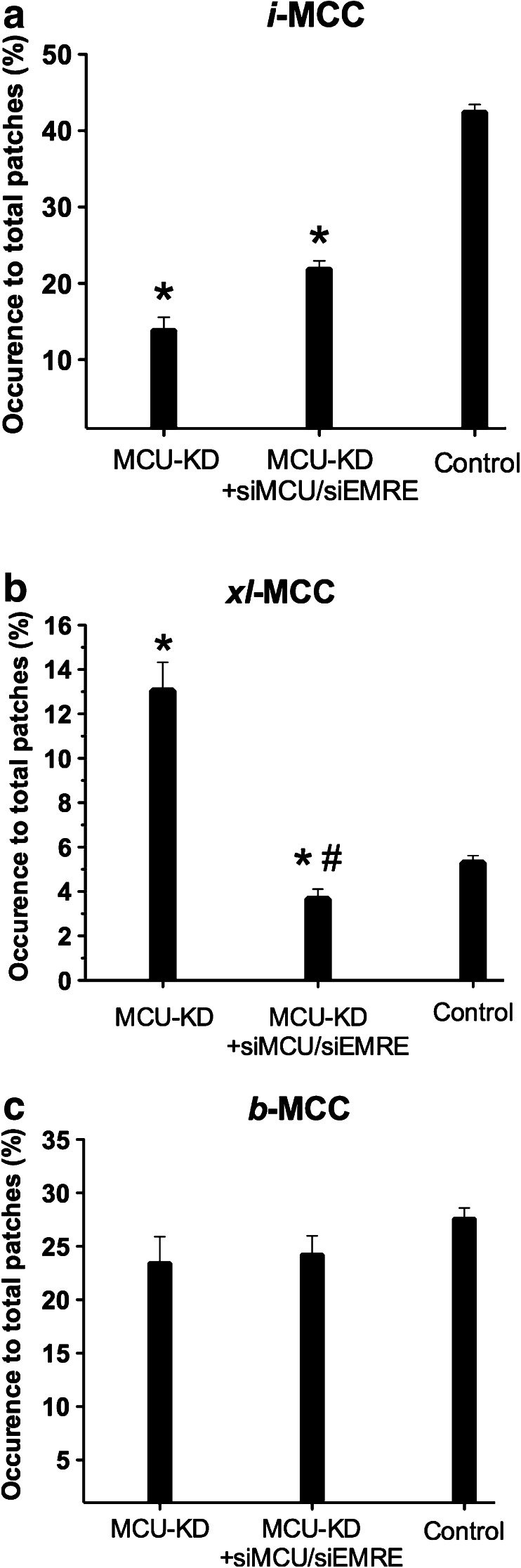
Statistical representation of mean values of occurrence of distinct single-channel activities in control, MCU-KD and MCU-KD, MCU-KD group treated with siRNA against MCU and EMRE (MCU-KD + siMCU + siEMRE). **a** *Bars* show the mean values of occurrence of *i*-MCC activity in MCU-KD. MCU-KD + siMCU + siEMRE and control groups. **b** Mean values of *xl-*MCC occurrence in MCU-KD. MCU-KD + siMCU + siEMRE and control groups. **c** Mean values of *b-*MCC occurrence in MCU-KD. MCU-KD + siMCU + siEMRE and control groups. *P > 0.05 vs. control, ^#^ *P* < 0.05 vs. MCU-KD

In MCU knockdown cells that were treated with siRNAs against MCU and EMRE, the occurrence of *i-*MCCs was 21.9 ± 4.3 % (22 patches out of 85 tested, N^D^ = 16) and similar (*P* = 0.19) to that 14.6 ± 6.0 % previously reported for MCU knockdown cells [4] and strongly reduced compared with the occurrence of *i-*MCC in control cells (42.5 ± 6.1 %, 52 out of 124 patches tested, N^D^ = 37; *P* < 0.05) (Fig. 4a).

In contrast to *i-*MCC, the occurrence of which was similar between MCU-KD cells and MCU-KD/siMCU-siEMRE cells, the occurrence of *xl-*MCC strongly decreased by treatment with siRNAs against MCU and EMRE from 13.1 ± 4.6 % (10 out of 67 patches tested, N^D^ = 13) in mitoplasts isolated from MCU-KD cells [4] to 3.7 ± 1.8 % (4 patches out of 85 tested, N^D^ = 16) (*P* < 0.05) (Fig. 4b).

Conductance of *i*-MCC and *xl*-MCC were not affected in mitoplasts isolated from MCU-KD cells [4] or MCU-KD/siMCU, siEMRE cells (*i*-MCC: control, 13.3 ± 0.8 pS, *n* = 14; MCU-KD/siMCU, siEMRE, 13.5 ± 0.9, *n* = 19, and, *xl*-MCC: control, 65.8 ± 4.6, *n* = 10; MCU-KD/siMCU, siEMRE, 66.5 ± 11.7 pS, *n* = 4).

The occurrence *b-*MCC was not altered by MCU and EMRE downregulation (control, 27.6 ± 6.1 %, N^D^ = 37; MCU-KD/siMCU-siEMRE, 24.2 ± 7.0 %, N^D^ = 16) (Fig. 4c).

### UCP2 knockdown has no effect on whole mitoplasts Ca^2+^ and Na^+^ currents

**Fig. 5:**
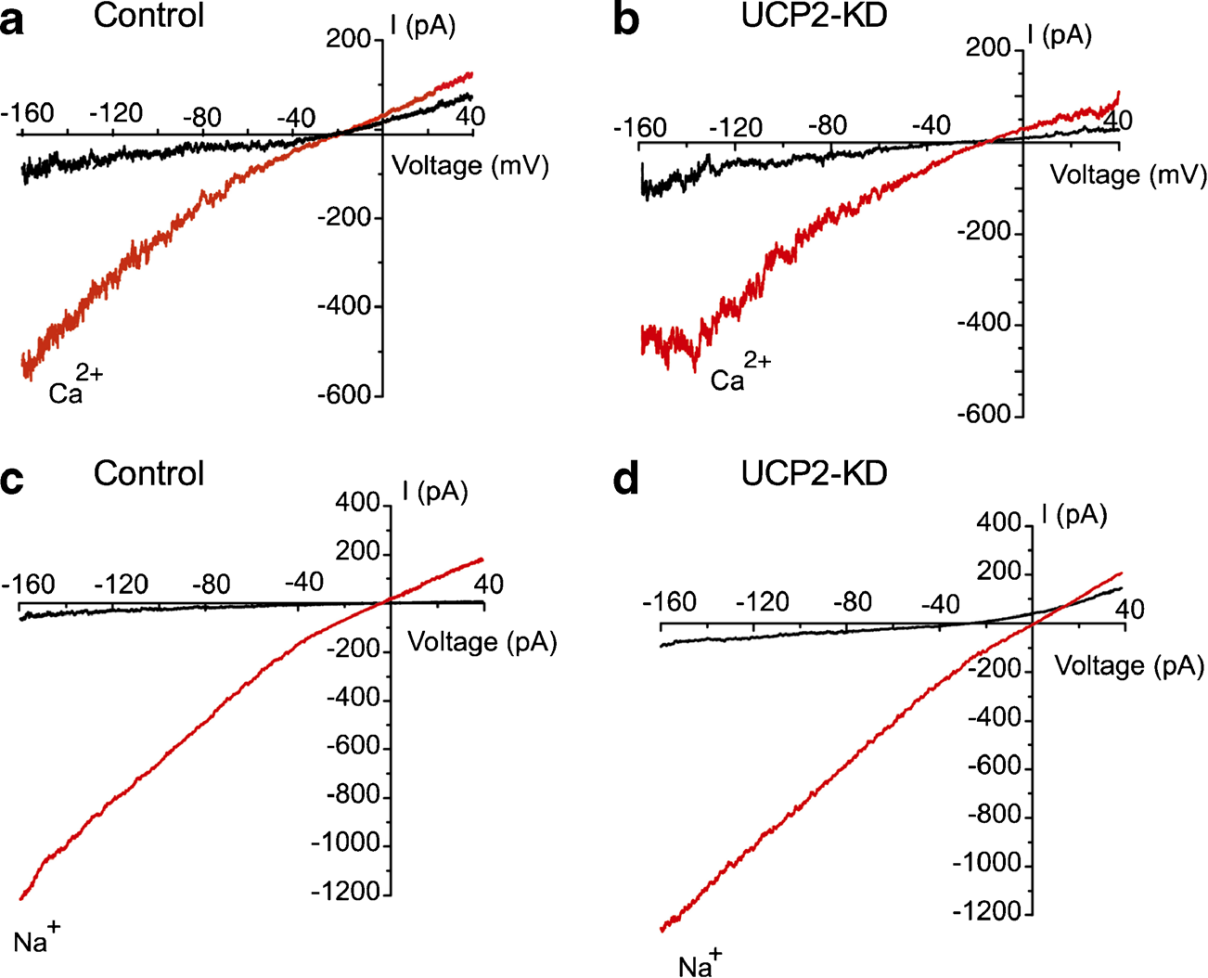
Effect of UCP2 diminution on whole-mitoplast cationic currents. **a** Representative whole-mitoplast current from mitoplast from control group prior (*black*) and after (*red*) addition of 3 mM Ca^2+^ to the bath. **b** Representative whole-mitoplast current from mitoplast from UCP2-KD group before (*black*) and after (*red*) addition of 3 mM Ca^2+^ to the bath. **c** Representative whole-mitoplast current from mitoplast from control group before (*black*) and after (*red*) addition of Na^+^ to divalent-free solution. **d** Representative whole-mitoplast current from mitoplast from UCP2-KD group before (*black*) and after (*red*) addition of Na^+^ to divalent-free solution (color figure online)

To assess a role of UCP2 in whole mitoplast Ca^2+^ fluxes, we assessed transmembrane Ca^2+^ currents in whole-mitoplast configuration. Addition of 3 mM Ca^2+^ into the bath solution during voltage ramps from −160 to 50 mV produced an inward current with the amplitude of 220.4 ± 43.5 pA (*n* = 15). UCP2 knockdown had no significant effect on transmitochondrial Ca^2+^ current (257.4 ± 42.5 pA, *n* = 23, Fig. 5a, b). Because in divalent-free conditions MCU is permeable for Na^+^, we also assessed whether Na^+^ current through MCU is altered when UCP2 expression was reduced. Similar to Ca^2+^ current, whole-mitoplast Na^+^ current was unaffected by UCP2 diminution (control, 659.6 ± 97.5 pA, *n* = 10; UCP2-KD, 644 ± 77.5 pA, *n* = 15) (Fig. 5c, d).

## Discussion

We have previously described that UCP2/3 are fundamentally involved in the activity of mitochondrial Ca^2+^ uptake under certain conditions [36]. Later studies described that the contribution of UCP2/3 to mitochondrial Ca^2+^ uptake is not ubiquitous [26] and requires yet unknown conditions [35] which might be due to the different activities of the mitochondrial Ca^2+^ uniporter in various tissues [13]. In subsequent studies, we described that, if UCP2/3 are involved in mitochondrial Ca^2+^ uptake, these proteins contribute to MCU-dependent mitochondrial Ca^2+^ influx [11] exclusively from intracellularly released Ca^2+^ [38, 40]. In contrast, sequestration of Ca^2+^ that enters the cell via the store-operated Ca^2+^ entry pathway was always independent from UCP2/3 but required essentially MCU and was facilitated by Letm1 [39]. These findings lead us to the assumption that UCP2/3, under yet unknown conditions, might serve as regulators of MCU/EMRE-dependent mitochondrial Ca^2+^ uptake pathway. Notably, the regulation of the activity of the MCU/EMRE-dependent pore is a pivotal step to regulate mitochondrial activity and to avoid mitochondrial Ca^2+^ overload that would yield initiation of the apoptotic cell death pathway. Accordingly, in view of its crucial importance for cellular activity and fate, a further modulation of MCU/EMRE-dependent mitochondrial Ca^2+^ uptake in addition to MICU1/MICU2 [9, 21, 22, 25, 41, 42] by UCP2/3 is feasible. Therefore, the present study was designed to challenge the concept of UCP2 being a modulator of MCU/EMRE-establish pore of the mitochondrial Ca^2+^ uniporter complex. Importantly, since the potential impact of UCP2/3 on mitoplast Ca^2+^ currents has never been evaluated in mitoplasts isolated from cells that exhibited UCP2/3 dependency in their mitochondrial Ca^2+^ uptake, in this study, mitoplasts were isolated from HeLa cells that have been described to exhibit UCP2/3 dependency in mitochondrial Ca^2+^ uptake [36, 37] and are well defined in this particular cell type [3, 4].

Because in our experiments with mitoplasts of UCP2-overexpressing cells no additional Ca^2+^ current besides *i-*MCC, *b-*MCC, and *xl-*MCC was observed, the formation of Ca^2+^ permeable channels by UCP2 alone can be excluded. However, overexpression of UCP2 strongly reduced the occurrence of *i-*MCC by approx. 44 %, while the occurrence of *xl-*MCC increased by approx. 3-fold and the appearance of *b-*MCC remained unchanged. These data indicate that a strong elevation of UCP2 favors the formation of *xl-*MCC on cost of the appearance of *i-*MCC, thus pointing to some exclusive commonalities between *i-*MCC and *xl-*MCC but not *b-*MCC. Because a strong knockdown of MCU/EMRE strongly reduced the *i-*MCC and *xl-*MCC occurrence but not that of *b-*MCC, we speculate that *i-*MCC and *xl-*MCC share/compete for MCU and EMRE. Thus, these findings provide evidence for two MCU/EMRE-dependent mitoplast Ca^2+^ channels (i.e., *i-*MCC and *xl-*MCC) and one MCU/EMRE-independent (i.e., *b-*MCC) mitoplast Ca^2+^ channel in one given cell type. The existence of various current densities of MCU-dependent Ca^2+^ currents has previously being reported in various tissues and has been discussed as to reflect the variability of mitochondrial Ca^2+^ uptake to meet the demand of the individual cell type [13].

Notably, in contrast to a strong knockdown of MCU/EMRE that strongly reduced the occurrence of *i-*MCC and *xl-*MCC, a moderate knockdown of MCU strongly affected the occurrence of *i-*MCC but not that of *xl-*MCC [4]. Accordingly, one can assume that the contribution of MCU to *xl-*MCC activity is more persistent that than that to *i-*MCC. Hence, our findings further indicate that UCP2 facilitates the formation of *xl-*MCC over *i-*MCC, although a direct interaction of UCP2 with MCU was not found in a very sophisticated proteomic assay [32]*.* Overall, despite the lack of the obvious effect of UCP2 downregulation on whole-mitoplast current, these data indicate that UCP2 favors the occurrence of *xl-*MCC that competes very efficiently, at least in the experimental setup of isolated mitoplasts from HeLa cells, with the dominantly established *i-*MCC for MCU, while a direct interaction between these two proteins can be excluded.

Considering that all data on Ca^2+^ currents/channels of the inner mitochondrial membrane have been conducted in artificial systems (either isolated mitoplasts or reconstituted membranes), it is still unclear whether any or, which of the reported Ca^2+^ currents (MicCa1/2 [6, 23]; mCa1/2 [24] or the MCCs [3] reflects that of intact cells. The data presented herein meet the common strategy of identification of the physiological relevance of mitoplast Ca^2+^ currents (i.e., sensitivity to ruthenium red; MCU/EMRE dependency) and provide evidence for the existence of an UCP2-regulated, MCU/EMRE-dependent, and ruthenium red-sensitive mitochondrial Ca^2+^ channel in intact cells that can be observed in isolated mitoplast also. Under which circumstances this channel gets involved in mitochondrial Ca^2+^ uptake remains unresolved and awaits further investigations.

In line with this positive influence on the occurrence of *xl-*MCC, UCP2 overexpression and knockdown yielded an almost 3-fold increase and approx. 38 % decreased open probability (NPo), respectively. Since no effect on the NPo of *i-*MCC by UCP2 overexpression or knockdown was found, these data describe UCP2 as selective modulator of one distinct, MCU-dependent, extra large conducting (~80 pS) mitoplast Ca^2+^ channel. In view of existing data that exclude a direct interaction between UCP2 and MCU [32], the actual mechanism of UCP2-exhibited modulatory effect on *xl-*MCC requires further investigation. It is important to note that single-channel activity was expressed as NPo, the product of the number of channels in the patch during recording (N) and the open channel probability (Po). Because in our experimental conditions (mitoplast-attached configuration) the precise number of active channels (N) is difficult to determine, the difference in mean NPo values may reflect either the increased open probability (Po), the number of active channels (N), or both. So, we cannot discard a possibility that upregulation in UCP2 protein increases the *xl*-MCC activity by enhancing the number of channels in the patches, while at the same time removing clusters of *i*-MCC from the membrane, leaving its individual cluster size unchanged. Collectively, these data represent the first demonstration of regulation of the biophysical characteristics (NPo) of a MCU/EMRE-dependent Ca^2+^ current in mitoplasts by a protein regulator.

Despite the significant influence of UCP2 on the activity of *xl-*MCC, no impact of diminution of UCP2 (UCP-KD) was found on whole mitoplast Ca^2+^/Na^+^ currents. Considering the small occurrence/contribution of *xl*-MCC versus *i-*MCC, one might expect a rather inconspicuous contribution of alterations in the activity of *xl-*MCC to whole mitoplast permeability to divalent cations. However, this assumption is in contradiction to our previous findings on a strong impact of UCP2 knockdown measured on mitochondrial Ca^2+^ uptake in intact cells of this very cell line [11, 36, 38, 40] that are perfectly in line with our present data on the regulatory role of UCP2 on *xl-*MCC. While the reason of these controversy remains to be further investigated, one need to admit that caution is necessary while extrapolating data from mitoplasts to the situation in intact cells. However, the present findings clearly point out that the ultimate answer which mitoplast Ca^2+^ current actually represents the mitochondrial Ca^2+^ uniporter in intact cells/tissue awaits final clarification.

The present study provides evidence of two biophysically distinct MCU/EMRE-dependent mitoplast Ca^2+^ channels of one given cell type. While the *i-*MCC is much more abundant than *xl-*MCC, MCU appears to have higher contribution to the latter one. Hence, an exclusive regulator function of UCP2 on *xl-*MCC is described, thus presenting UCP2 as governor of one distinct mitochondrial Ca^2+^ current besides the ubiquitously acting MICU1/2.
